# Observations on the Projected Bill for Restricting the Practice of Surgery and Midwifery to the Members of the Royal Colleges of London, Edinburgh, and Dublin, &c.

**Published:** 1817-01

**Authors:** 


*Observations on the projected Bill for restricting the Practice of Surgery and Midwifery to the Members of the Royal Colleges of London, Edinburgh, and Dublin, &c.* By a General Practitioner, 8vo. pp. 31. London, 1816.

THERE is a good deal of sound sense and strong argument in this pamphlet. In the celebrated attempt at Medical Reform, headed by Sir Joseph Banks, it was proved, that in most parts of Great Britain, the proportion of regular to irregular practitioners, was very small—an average of only one in five or six! Now, our author justly asks, how an increase of *expence* is likely to augment the number of the educated? Indeed, we think the idea of attaching a large fee for examination is very preposterous, to say the least of it. If there be not virtue or honesty enough in colleges and courts to make a rigid enquiry into the acquirements of candidates for diplomas, without any pecuniary considerations, there is little chance of purging the profession of ignorance and incompetency. We do not quite agree with our author, however, respecting the *increased attendance* on hospitals and lectures, which is contemplated in the Bill. This measure we think judicious in general, though we are of opinion, that the youth of genius and industry should not be debarred the liberty of presenting himself for examination, as soon as the *original term* of study is expired—thus making the acquirements, and not the time of acquiring, the *sine qua non* of honorary degrees.

"Is then the assiduous, studious, and well informed pupil to suffer—to be excluded from a profession in which he must be useful, and to which he may be an ornament, because some of his brother students, idle, dissolute, or without talent, require double the time and pecuniary expenditure for equal sufficiency."—P. 14.

We agree with the writer of this pamphlet, that, any measure reducing the number of competent practitioners below what the exigencies of the public require, and calling for remuneration beyond what the ordinary emoluments of practice afford, must defeat its very object. Any ultra-restrictive proposition, even if it becomes law, cannot long have being and effect. No legislative enactments can prevent the people throwing themselves into the delusive arms of ignorance and quackery in their misfortunes, if educated and proper advice is placed beyond their reach and command.

"In concluding these observations, the author wishes to observe that, as he has no college honours to seek, the question of expence for their diploma is not to him one of pecuniary interest: but, no stranger to calamity in his early days himself, he cannot be otherwise than feelingly alive to the hard fate of some (perhaps of many) of the present students in London; who, he well knows, as orphans, with misfortune for their patrimony, from the pressure of the times,—and the "*Res angusta Domi*,"—cannot, under any circumstances, prolong their stay and studies beyond the present winter; and who, consequently, if this bill becomes "law," *debarred from even approaching the probationary tribunal*, must return into the country to see empiricism in its various shapes flourish, while they are cut off from the profession of surgery and midwifery, for which they have been educated—and from which they have been led to expect a livelihood, honourable to themselves, and useful to the community." P. 30.

We recommend the pamphlet to the perusal of those interested in the subject, or capable of influencing the decision of the question.

